# Metabolic Profiling Analysis of the Alleviation Effect of the Fractions of Niuhuang Jiedu Tablet on* Realgar* Induced Toxicity in Rats

**DOI:** 10.1155/2018/2154603

**Published:** 2018-01-23

**Authors:** Wenfeng Xu, Yuehu Pei, Shuo Xu, Haifeng Wang, Pengfei Jin

**Affiliations:** ^1^Department of Pharmacy, National Center of Gerontology, Beijing Hospital, Beijing 100730, China; ^2^School of Traditional Chinese Materia Medica, Shenyang Pharmaceutical University, Shenyang 110016, China; ^3^Key Laboratory of Structure-Based Drug Design and Discovery, Ministry of Education, Shenyang Pharmaceutical University, Shenyang 110016, China

## Abstract

Niuhuang Jiedu Tablet (NJT) is a classical formula in treating acute tonsillitis, pharyngitis, and so on. In the formula, significant level of* Realgar *as a potentially toxic element is contained. Our previous experiments revealed that it was less toxic for combined* Realgar *in NJT. However, the active fraction of this prescription with toxicity alleviation effect on* Realgar *was still obscure. NJT was divided into five different polar fractions (NJT-PET, NJT-25, NJT-50, NJT-75, and NJT-95), and we explored the toxicity alleviation effect on* Realgar*. Based on ^1^H NMR spectra of urine and serum from rats, PCA and PLS-DA were performed to identify different metabolic profiles. Liver and kidney histopathology examinations and serum clinical chemistry analysis were also performed. With pattern recognition analysis of metabolites in urine and serum, Realgar group showed a clear separation from control group, while the metabolic profiles of NJT-PET, NJT-25, NJT-50, and NJT-95 groups were similar to Realgar group, and the metabolic profiles of NJT and NJT-75 groups were very close to control group. Statistics results were confirmed by the histopathological examination and biochemical assay. The present work indicated that 75% EtOH fraction of NJT was the most valid fraction with the toxicity alleviation effect on* Realgar*.

## 1. Introduction

*Realgar* (As_2_S_2_), as a type of mineral drug that contains arsenic, has been used in traditional Chinese medicines (TCMs) for thousands of years. It has been used for the treatment of carbuncles, boils, insect and snake bites, intestinal parasitosis, convulsive epilepsy, and psoriasis [1]. Rather than being used alone,* Realgar* is commonly prescribed with other herbal medicines or minerals in Chinese compound formulae. Niuhuang Jiedu Tablet (NJT) composed of* Realgar* and seven other TCMs is one of the most commonly used over-the-counter TCMs for the treatment of acute tonsillitis, pharyngitis, periodontitis, and mouth ulcer. Each tablet contains 3.33 mg of* Bovis Calculus Artifactus*, 33.3 mg of* Realgar*, 133.2 mg of* Gypsum Fibrosum*, 16.7 mg of* Borneolum Syntheticum*, 133.2 mg of* Rhei Radix et Rhizoma*, 99.9 mg of* Scutellariae Radix*, 66.6 mg of* Platycodonis Radix,* and 33.3 mg of* Glycyrrhizae Radix et Rhizoma* [2]. Arsenic is well known for its acute and chronic toxicity [3]. Although* Realgar* is poorly soluble in water and considered to be less poisonous, upon ingestion of* Realgar*-containing TCMs, the potential of arsenic poisoning cannot be ignored [4]. Recently, studies on the acute and chronic toxicological effects and poisoning cases of* Realgar* have been reported [1, 3, 5]. The safety of NJT is of concern.

Metabonomics is defined as “the quantitative measurement of the dynamic multiparametric metabolic response of living systems to pathophysiological stimuli or genetic modification” [6]. It can isolate potential biomarkers and metabolic networks by measuring and mathematically modeling the changes in the metabolism product levels in biological fluids and tissues [7]. The integral and systematic study of metabonomics is in agreement with TCMs theory in nature and the metabonomics may be the best approach to fit the holistic concept of TCMs. Therefore, the introduction of the concept of metabonomics offers great and novel opportunities to reinvestigate TCMs [8]. ^1^H NMR, which is noninvasive and nondestructive, reveals the overall metabolic profile of biofluids or tissue extracts and offers vital information on metabolite structure [9]. NMR-based metabonomics have been widely applied in TCMs toxicity research [10].

In our previous study, the toxicity of* Realgar* combined with other TCMs in NJT was investigated, and the results indicated that it was more secure and less toxic for combined* Realgar* in NJT [11]. However, the active fraction of this prescription in producing toxicity alleviation effect on* Realgar* was still obscure. The objective of the present work was to evaluate the toxicity alleviation effect of five different polar fractions of NJT on* Realgar* using metabonomics method based on NMR.

## 2. Experimental

### 2.1. Material and Reagents

D_2_O (deuterium oxide, 99.8% in D) and sodium-3-(trimethylsilyl) propionate-2, 2, 3, 3-d_4_ (TSP) were purchased from Norell Inc. (USA). All the other chemicals were of analytical grade and commercially available.* Realgar*,* Bovis Calculus Artifactus*,* Borneolum Syntheticum*,* Gypsum Fibrosum*,* Rhei Radix et Rhizoma*,* Scutellariae Radix*,* Platycodonis Radix,* and* Glycyrrhizae Radix et Rhizoma* were purchased from Anguo Changda Chinese Herbal Pieces Ltd. (Anguo, China) and identified by Professor Yuehu Pei (Shenyang Pharmaceutical University, China). The voucher specimens were deposited in the School of Traditional Chinese Materia Medica of Shenyang Pharmaceutical University.

### 2.2. Preparation of the Extracts

**Figure 1:**
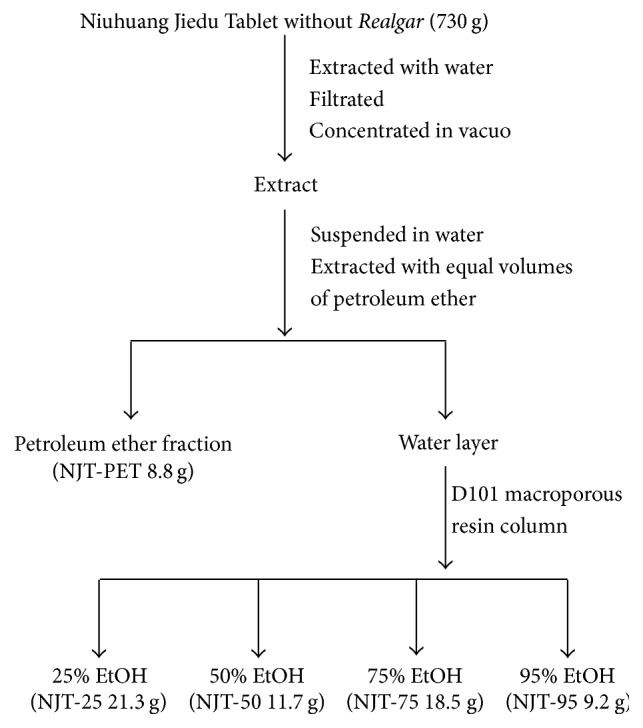
Flow diagram of fractionations of the Niuhuang Jiedu Tablet prescription.

A 730 g amount of mixed herbs (NJT without* Realgar*), that is,* Bovis Calculus Artifactus*,* Borneolum Syntheticum*,* Gypsum Fibrosum*,* Rhei Radix et Rhizoma*,* Scutellariae Radix*,* Platycodonis Radix,* and* Glycyrrhizae Radix et Rhizoma* in the weight ratio of 0.1 : 0.5 : 4 : 4 : 3 : 2 : 1, was extracted with water (4 L) under reflux for 2 h, a process that was repeated twice. After filtration, the extracts were combined and concentrated by vacuum evaporation to syrup, which was suspended in water (800 mL). The suspension was extracted with equal volumes of petroleum ether, and the petroleum ether fraction (NJT-PET) was obtained (8.8 g). The water layer was further chromatographed over a D101 macroporous resin column, eluted with 25%, 50%, 75%, and 95% ethanol gradually and the fractions NJT-25, NJT-50, NJT-75, and NJT-95 were obtained (21.3 g, 11.7 g, 18.5 g, and 9.2 g), respectively. Preparation of the extract and derived fractions is illustrated in Figure 1.

### 2.3. Animals

Forty-eight male Wistar rats (weighing 200 ± 20 g) were purchased from Experimental Animal Center of Shenyang Pharmaceutical University (no. SCXK (Liao) 2010-0001). During the whole experiment procedure, the rats were maintained in a 12 h/12 h light/dark cycle at a constant temperature of 22 ± 3°C with relative humidity of 45–60% and allowed free access to food and water. Animal studies were conducted under approved guidelines of the Animal Ethics Committee of Shenyang Pharmaceutical University.

### 2.4. Drug Administration and Sample Collection

All drugs were dispersed in 1% CMC-Na. After one-week acclimatization, the rats were randomly distributed into eight groups of six each: (1) control group (equivalent volumes of 1% CMC-Na), (2) Realgar group (*Realgar* 2 g/kg), (3) NJT group (Niuhuang Jiedu Tablet 10 g/kg), (4) NJT-PET group (coadministration of NJT-PET 352 mg/kg and* Realgar* 2 g/kg), (5) NJT-25 group (coadministration of NJT-25 852 mg/kg and* Realgar* 2 g/kg), (6) NJT-50 group (coadministration of NJT-50 468 mg/kg and* Realgar* 2 g/kg), (7) NJT-75 group (coadministration of NJT-75 740 mg/kg and* Realgar* 2 g/kg), and (8) NJT-95 group (coadministration of NJT-95 368 mg/kg and* Realgar* 2 g/kg). Animals were given the calculated amounts of materials via gastric intubation one time each day for consecutive seven days with the administration of volume of 20 mL/kg (rat body weight).

Urine samples were collected in tubes containing 50 *μ*L sodium azide (1%, w/v) over ice packs from 8:00 p.m. to 8:00 a.m. on predose day −1 and on postdose days one to eight. The collected urine samples were stored at −20°C until analysis. Rats were sacrificed on day eight, and blood samples drawn from abdominal aorta were centrifuged at 15000 ×g for 10 min at 4°C to obtain serum samples. All serum samples were frozen at −80°C for further analysis. Livers and kidneys were rapidly isolated, washed in sterile 0.9% (w/v) sodium chloride solution, and fixed in 10% formalin solution for histopathological examination.

### 2.5. Clinical Biochemistry and Histopathology

Clinical biochemical parameters of serum included aspartate aminotransferase (AST), alanine aminotransferase (ALT), alkaline phosphatase (ALP), blood urea nitrogen (BUN), creatinine (CREA), triglyceride (TG), and total cholesterol (TC) were performed with an automated Hitachi Analyzer (Hitachi Medical Corporation, Tokyo, Japan).

Liver and kidney tissues were embedded in paraffin blocks, sectioned to 5 *μ*m thickness, and stained with hematoxylin and eosin (H + E) for histopathological evaluation.

### 2.6. ^1^H NMR Spectroscopic Measurement of Urine and Serum Samples

After thawing at room temperature, an aliquot of 400 *μ*L urine and 200 *μ*L phosphate buffer (0.2 M, pH 7.4) were mixed to minimize chemical shift variation. Then 500 *μ*L supernatants were obtained by centrifugation at 3500 ×g for 5 min and were transferred into 5 mm NMR tubes to which 40 *μ*L TSP (1 mg/mL) and 20 *μ*L D_2_O were added. TSP served as an internal chemical shift reference (*δ* 0.0) and D_2_O provided a field frequency lock-solvent for the NMR spectrometer. ^1^H NMR measurements of urine samples were recorded on a Bruker AV 600 MHz spectrometer at 298 K using one-dimensional (1D) Nuclear Overhauser Effect Spectroscopy (NOESY) pulse sequence with presaturation during relaxation delay (RD = 3.0 s) and mixing time (*t*_*m*_ = 0.1 s) to suppress the water signal. A total of 64 transients were collected into 64K data points with a spectral width of 12 kHz and an acquisition time of 2.73 s.

Serum samples were thawed at room temperature and centrifuged at 15000 ×g for 10 min. 300 *μ*L of the supernatant was transferred into 5 mm NMR tubes containing 100 *μ*L TSP (1 mg/ml) and 200 *μ*L D_2_O. NMR spectra of serum samples were also measured on a Bruker AV 600 MHz spectrometer. Pulse program was one-dimensional Carr-Purcell-Meiboom-Gill (CPMG) sequence. 64 free induction decays (FIDs) were collected into 64k data points using a spectral width of 12 kHz and an acquisition time of 2.73 s.

### 2.7. NMR Spectral Data Reduction and Pattern Recognition

All ^1^H NMR spectra were manually phased, baseline adjusted, and referenced to TSP at *δ* 0.00 using MestReNova 9.0.1 software (Mestrelab Research SL). Every spectrum was subsequently segmented into regions at 0.04 ppm intervals across the chemical shift *δ* 9.60–0.20. The regions of *δ* 5.20–4.72 in urine spectra and *δ* 5.20–4.60 in serum spectra were excluded to avoid any baseline distortion caused by imperfect water suppression. The regions of *δ* 6.20–5.48 in urine spectra were also removed to eliminate the influence of broad resonance from urea. The regions contributed to citrate (*δ* 2.70–2.64 and *δ* 2.58–2.52) were combined into two signals *δ* 2.66 and *δ* 2.54, respectively. The integrated data were normalized to the total integrals of each spectrum to compensate for the concentration differences.

The mean-centered NMR data were submitted to SIMCA-P 11.5 software package (Umetrics AB, Umea, Sweden) for pattern recognition analysis. Principal component analysis (PCA) was initially used to obtain a general overview of the metabolic pattern. Then partial least square discriminant analysis (PLS-DA) was performed to reveal the different metabolic alterations between the dosed and control group. The quality of these models can be explained by *R*^2^ and *Q*^2^ values. *R*^2^ is used to evaluate the fitting condition of the models and *Q*^2^ is used to assess the predictive ability. Data were visualized with the scores plots and loadings plots. Each point on scores plots represents an individual spectrum of a sample and each point on loadings plots represents a single spectral region or chemical shifts. Classification of samples and the endogenous metabolites responsible for the classification can be shown from the scores and loadings plots, respectively.

### 2.8. Statistical Analysis

All numerical data are presented as mean ± SD. The data were statistically analyzed through one-way analysis of variance (one-way ANOVA) and Dunnett *t*-test using SPSS 16.0 software (Chicago, Inc., USA). Values of *P* < 0.05 were considered to be statistically significant.

## 3. Results

### 3.1. Clinical Biochemistry and Histopathology

As shown in Table 1, statistically significant increased serum levels of AST, ALT, ALP, BUN, and CREA were observed in Realgar group compared with control group. Combined with NJT and NJT-75,* Realgar* did not exert significant effects on serum clinical chemistry parameters. Serum enzyme values of the rest groups were elevated in varying degrees compared to the control group.

**Figure 2:**
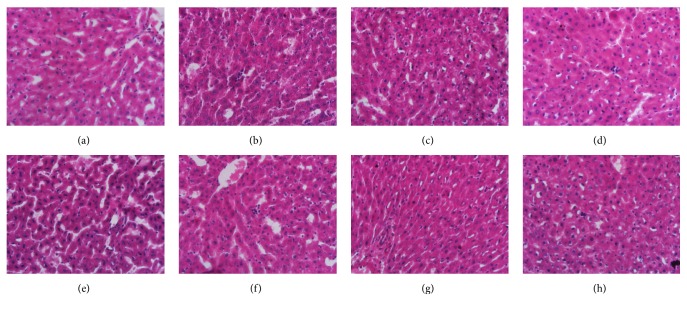
Photomicrographs of representative sections of livers from the control group (a), Realgar group (b), NJT group (c), NJT-PET group (d), NJT-25 group (e), NJT-50 group (f), NJT-75 group (g), and NJT-95 group (h).

**Figure 3:**
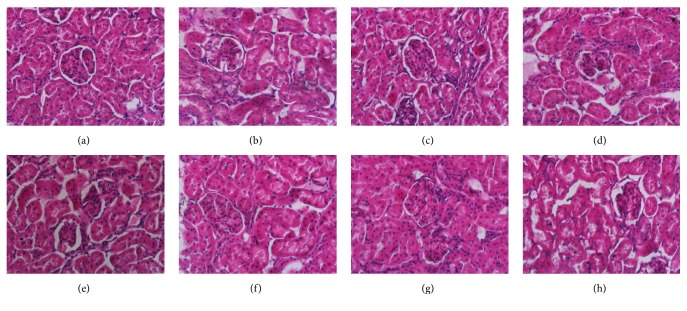
Photomicrographs of representative sections of kidneys from the control group (a), Realgar group (b), NJT group (c), NJT-PET group (d), NJT-25 group (e), NJT-50 group (f), NJT-75 group (g), and NJT-95 group (h).

Histopathological findings of livers and kidneys of rats from the eight groups were displayed in Figures 2 and 3. There were no signs of apparent abnormality observed in control livers and kidneys. Slight necrosis, swelling of hepatocytes, and mild tubular lesions of renal cortex were observed in rats treated with* Realgar*. Rats of NJT and NJT-75 group showed recovery trends for liver and kidney injuries induced by* Realgar*.

### 3.2. Analysis of ^1^H NMR Spectra of Urine

**Figure 4:**
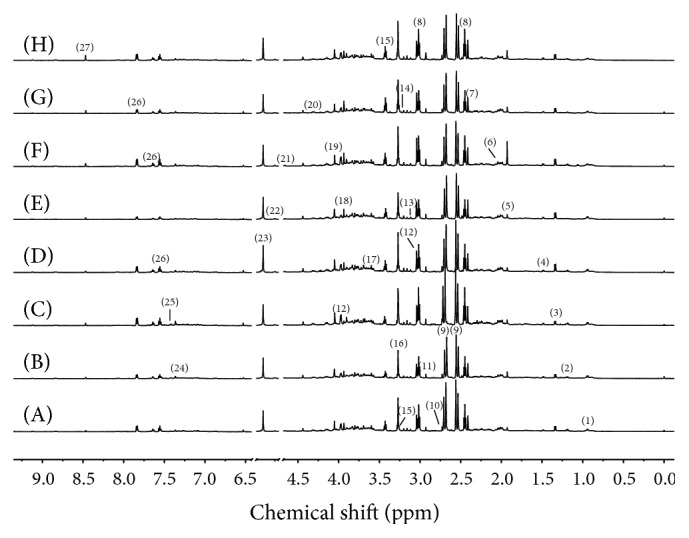
^1^H NMR spectra of urine samples of rats from the control group (A), Realgar group (B), NJT group (C), NJT-PET group (D), NJT-25 group (E), NJT-50 group (F), NJT-75 group (G), and NJT-95 group (H). (1) Leucine + isoleucine; (2) 3-hydroxybutyrate; (3) lactate; (4) alanine; (5) acetate; (6) acetoacetate; (7) pyruvate; (8) 2-ketoglutarate; (9) citrate; (10) dimethylamine; (11) dimethylglycine; (12) creatine; (13) malonate; (14) choline; (15) taurine; (16) trimethylamine-N-oxide; (17) glycine; (18) betaine; (19) creatinine; (20) malate; (21) *β*-glucose; (22) *α*-glucose; (23) allantoin; (24) phenylalanine; (25) 3-indoxylsulfate; (26) hippurate; (27) formate.

Typical ^1^H NMR spectra of urine samples from each group were shown in Figure 4. The metabolite resonances were assigned according to previous studies [11–14] and our preexperiments. Twenty-seven metabolites including leucine/isoleucine, 3-hydroxybutyrate, lactate, alanine, acetate, acetoacetate, pyruvate, 2-oxoglutarate, citrate, dimethylamine, dimethylglycine, creatine, malonate, choline, taurine, trimethylamine-N-oxide (TMAO), glycine, betaine, creatinine, malate, *β*-glucose, *α*-glucose, allantoin, phenylalanine, 3-indoxylsulfate, hippurate, and formate were detected.

**Figure 5:**
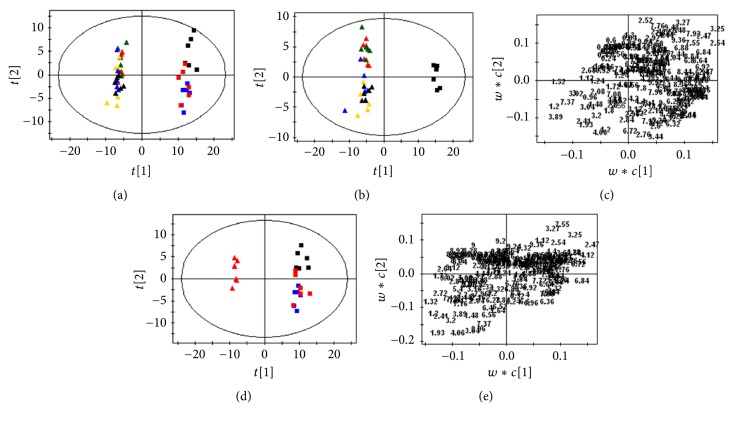
PCA scores plot (a) derived from ^1^H NMR spectra of urine from the eight groups (*R*^2^*X* = 0.827, *Q*^2^ = 0.659). PLS-DA scores plot and corresponding loadings plot based on ^1^H NMR spectra of urine from control group, Realgar group, NJT-PET group, NJT-25 group, NJT-50 group, and NJT-95 group ((b) and (c) *R*^2^*Y*-intercept of 0.375, *Q*^2^*Y*-intercept of −0.213) and from control group, Realgar group, NJT group, and NJT-75 group. ((d) and (e) *R*^2^*Y*-intercept of 0.334, *Q*^2^*Y*-intercept of −0.191). Control group (black square), Realgar group (red triangle), NJT group (blue square), NJT-PET group (green triangle), NJT-25 group (yellow triangle), NJT-50 group (blue triangle), NJT-75 group (red square), and NJT-95 group (black triangle).

PCA-based profiling of the NMR spectra of the urine samples from the eight groups was employed to explore the intrinsic differences in the metabolisms of these rats (Figure 5(a)). The samples from different groups were classified. The points of NJT and NJT-75 groups are distributed in the area of control group, while groups NJT-PET, NJT-25, NJT-50, and NJT-95 cluster very closely to Realgar group.

In order to maximize the separation between experimental groups and to focus on metabolic variations significantly contributing to classifications, the PLS-DA models were subsequently performed. As shown in the scores plot (Figure 5(b)), the separation of Realgar, NJT-PET, NJT-25, NJT-50, and NJT-95 groups from control group is clearly seen, indicating that they had different metabolic profiles from control group. A loadings plot is generated to identify the metabolites responsible for the differentiation in the scores plot. It shows increased levels of leucine, isoleucine, 3-hydroxybutyrate, lactate, alanine, acetate, pyruvate, creatine, choline, taurine, betaine, creatinine, and phenylalanine with decreased levels of 2-oxoglutarate, citrate, TMAO, and hippurate in Realgar, NJT-PET, NJT-25, NJT-50, and NJT-95 groups compared with control group (Figure 5(c)).

The PLS-DA scores plot reveals that Realgar group could be easily distinguished from control, NJT, and NJT-75 groups (Figure 5(d)). From examination of the corresponding loadings plot and NMR spectra, the separation is attributed to the depletion of leucine, isoleucine, 3-hydroxybutyrate, lactate, alanine, acetate, pyruvate, creatine, choline, taurine, betaine, and phenylalanine with elevated 2-oxoglutarate, citrate, TMAO, and hippurate in NJT and NJT-75 groups compared with Realgar group (Figure 5(e)).

**Figure 6:**
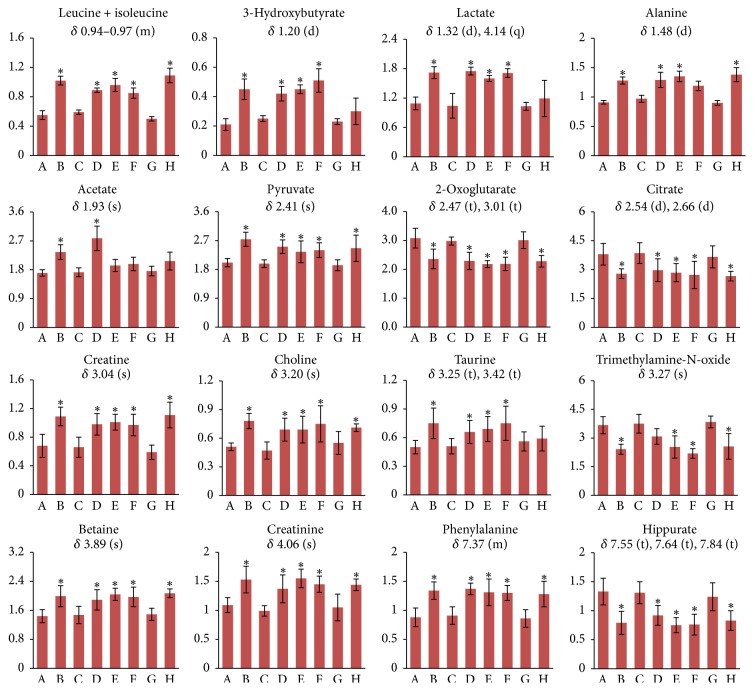
^1^H NMR-detected relative integral levels of endogenous metabolites in urine samples of the control group (A), Realgar group (B), NJT group (C), NJT-PET group (D), NJT-25 group (E), NJT-50 group (F), NJT-75 group (G), and NJT-95 group (H). Data are presented as mean ± SD of six animals per group. Multiplicity: s, single; d, double; t, triplet; q, quartet; and m, multiplet. ^*∗*^*P* < 0.05 versus control group.

The ^1^H NMR-detected relative integral levels of metabolites in urine samples of different groups are shown in Figure 6.

### 3.3. Analysis of ^1^H NMR Spectra of Serum

**Figure 7:**
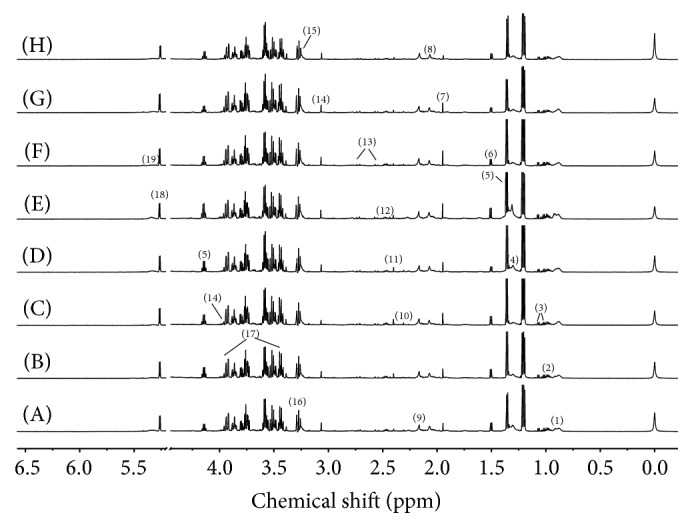
^1^H NMR spectra of serum samples of rats from control group (A), Realgar group (B), NJT group (C), NJT-PET group (D), NJT-25 group (E), NJT-50 group (F), NJT-75 group (G), and NJT-95 group (H). (1) Very low-density lipoprotein (VLDL)/low-density lipoprotein (LDL)-CH_3_; (2) leucine + isoleucine; (3) valine; (4) VLDL/LDL-CH_2_-; (5) lactate; (6) alanine; (7) acetate; (8) N-acetyl glycoprotein; (9) methionine; (10) acetoacetate; (11) pyruvate; (12) 2-ketoglutarate; (13) citrate; (14) creatine; (15) choline; (16) trimethylamine-N-oxide; (17) glucose; (18) *α*-glucose; and (19) unsaturated lipid.

Figure 7 shows representative 600 MHz ^1^H NMR spectra of serum from controls and treated groups. Assignments of endogenous metabolites were based on the literatures [15, 16] and our preexperiments. Nineteen metabolites were investigated and characterized as very low-density lipoprotein (VLDL), low-density lipoprotein- (LDL-) CH_3_, leucine/isoleucine, valine, VLDL/LDL-CH_2_, lactate, alanine, acetate, N-acetyl glycoprotein, methionine, acetoacetate, pyruvate, 2-ketoglutarate, citrate, creatine, choline, TMAO, glucose, and unsaturated lipid.

**Figure 8:**
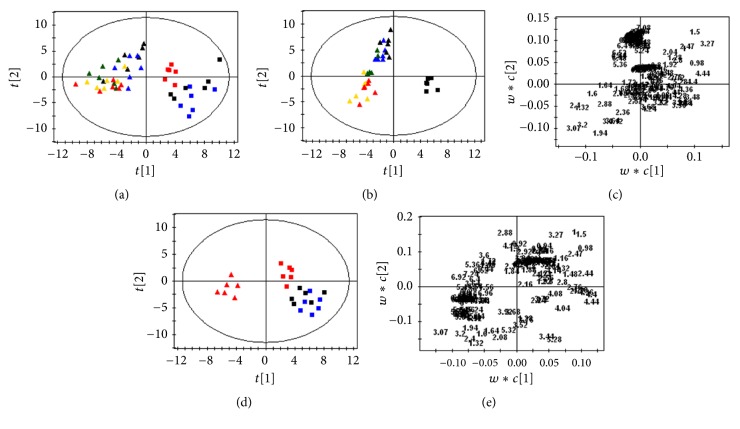
PCA scores plot (a) derived from ^1^H NMR spectra of serum from the eight groups (*R*^2^*X* = 0.742,* Q*^2^ = 0.616). PLS-DA scores plot and corresponding loadings plot based on ^1^H NMR spectra of serum from control group, Realgar group, NJT-PET group, NJT-25 group, NJT-50 group, and NJT-95 group ((b) and (c)* R*^2^*Y*-intercept of 0.311,* Q*^2^*Y*-intercept of −0.275) and from control group, Realgar group, NJT group, and NJT-75 group ((d) and (e)* R*^2^Y-intercept of 0.381,* Q*^2^*Y*-intercept of −0.304). Control group (black square), Realgar group (red triangle), NJT group (blue square), NJT-PET group (green triangle), NJT-25 group (yellow triangle), NJT-50 group (blue triangle), NJT-75 group (red square), and NJT-95 group (black triangle).

PCA was firstly conducted for the eight groups, and all the spots are located inside of the confidence interval (Figure 8(a)). An obvious separation between Realgar group and control group is observed. The points of groups NJT and NJT-75 are classified to the region of the controls, while Realgar, NJT-PET, NJT-25, NJT-50, and NJT-95 group are mapped together.

To further identify the distinction of the metabolic profiles and discriminating metabolites, PLS-DA was applied to the data. The scores plot (Figure 8(b)) shows that samples of Realgar, NJT-PET, NJT-25, NJT-50, and NJT-95 group are well discriminated from control group. The metabolites responsible for the significant separation between the groups are identified in the corresponding loadings plot (Figure 8(c)). According to the criterion of the loadings plot, the elevation in the levels of lactate, acetate, pyruvate, creatine, and choline and a reduction in the levels of leucine, isoleucine, valine, alanine, 2-oxoglutarate, and TMAO in Realgar, NJT-PET, NJT-25, NJT-50, and NJT-95 groups compared with control group contribute to the separation.

The performance of scores plot reflects discrimination where NJT and NJT-75 groups lie apart from Realgar group (Figure 8(d)). The major biochemical changes identified in serum from the corresponding loadings plot (Figure 8(e)) and ^1^H NMR spectra are increased leucine, isoleucine, valine, alanine, 2-oxoglutarate, and TMAO with decreased lactate, acetate, pyruvate, creatine, and choline in NJT and NJT-75 groups compared with Realgar group.

**Figure 9:**
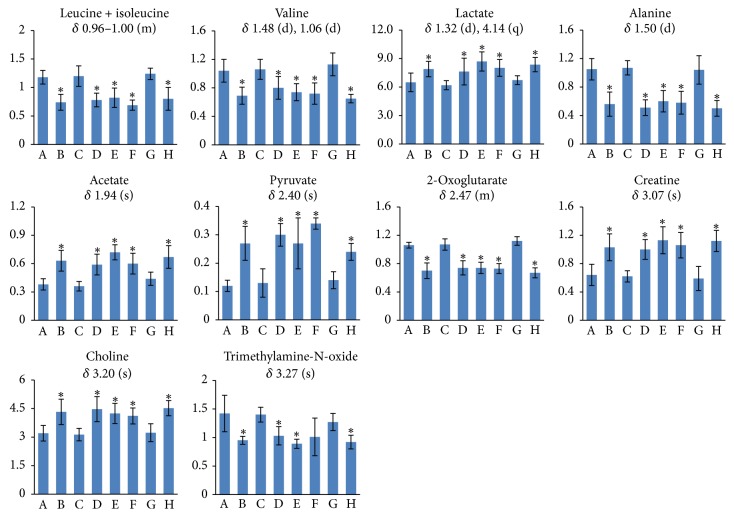
Alterations of relative integral concentrations of endogenous metabolites in serum from the control group (A), Realgar group (B), NJT group (C), NJT-PET group (D), NJT-25 group (E), NJT-50 group (F), NJT-75 group (G), and NJT-95 group (H). Data are presented as mean ± SD of six animals per group. Multiplicity: s, single; d, double; q, quartet; and m, multiplet. ^*∗*^*P* < 0.05 versus control group.

The ^1^H NMR-detected relative integral levels of metabolites in serum samples of different groups are shown in Figure 9.

## 4. Discussion

In our previous experiments, the toxicity alleviation effect of other TCMs in NJT on* Realgar* was verified [11], but the active fraction of NJT that has toxicity alleviation effect on* Realgar* was not yet clear. In the present tests, NJT was therefore divided into five fractions: NJT-PET, NJT-25, NJT-50, NJT-75, and NJT-95, to find the active fraction. Based on histopathology examinations, serum clinical chemistry analysis, and metabolomics method, NJT-75 was ascertained to be the active fraction of NJT that had toxicity alleviation effect on* Realgar*.

ALT and AST in liver cells can leak into serum during hepatic injury [17], and the measurements of these parameters are considered good indicators of liver cell damage [18]. Increasing level of ALP in serum might result from cholestasis, which may occur due to intrahepatic causes, extrahepatic obstruction, or infiltrative disorders of the liver [19]. BUN is the product of amino acids and CREA is the ultimate product of creatine metabolism in skeletal muscle. Concentrations of BUN and CREA are important indicators of renal function [20].

### 4.1. Protective Effect of Energy Metabolism of NJT and NJT-75

Tricarboxylic acid (TCA) cycle is a significant biological metabolic pathway that not only involves glucose aerobic oxidation but also participates in the major pathways of lipid oxidation and amino acid metabolisms [21]. One of the prominent findings was the decreases of the TCA cycle intermediates, including citrate and 2-oxoglutarate, in urine and serum samples from* Realgar*-dosed rats, which indicated that* Realgar* caused suppression of the TCA cycle and could further induce organ failure [13, 22, 23]. One possible cause of decreased levels of TCA cycle intermediates was that pyruvate dehydrogenase, a key enzyme converting pyruvate into acetyl-CoA, was inhibited [24]. This notion was supported by the observation of elevated levels of pyruvate in the urine and serum of* Realgar*-exposed rats.

Marked elevated level of plasma glucose found in the* Realgar*-dosed rats suggested that* Realgar* caused stimulated glycogenolysis and glycolysis, which makes contribution to the elevated levels of pyruvate [24]. Meanwhile, there was a correlated increase in lactate concentrations evident in the corresponding urine and serum, which offered further support for increased rates of glycogenolysis and glycolysis, since pyruvate could be converted to lactate via lactate dehydrogenase (LDH) in order to alternate to the less-efficient anaerobic respiration for energy production [25, 26].

Furthermore, levels of ketone bodies, such as acetate and 3-hydroxybutyrate, were increased in urine and serum samples of* Realgar*-dosed rats. These ketone bodies are the products of *β*-oxidation for fatty acid in mitochondria [27]. Elevations of acetate and 3-hydroxybutyrate suggested the promoted *β*-oxidation of fatty acid and the reduced utilization of acetyl-CoA into the TCA cycle or an increase in anaerobic cell respiration, which was a kind of energy metabolism process [28].

NJT-75 could recover the lower metabolic levels of citrate and 2-oxoglutarate, and the higher levels of pyruvate, lactate, acetate, and 3-hydroxybutyrate induced by* Realgar*, which suggested that NJT-75 had the same accommodation effects on the energy metabolism disorder as NJT.

### 4.2. Protective Effect of Choline Metabolism of NJT and NJT-75

Choline is a major membrane constituent and is important to the integrity of cell membranes and lipid metabolism [29]. Consistently higher concentrations of urine and serum choline are believed to be involved in membrane impairment [30], which is in agreement with previously reported enhanced membrane permeability and altered membrane structure by* Realgar* exposure [31], and the observed swelling and necrosis of hepatocytes as well as the elevated AST, ALT, and ALP in plasma biochemical examinations.

However, when* Realgar* treatment was combined with the administration of NJT and NJT-75, the concentration of choline got close to its normal level, which indicated that NJT and NJT-75 have protective effects on membrane impairment induced by* Realgar* treatment.

### 4.3. Balance Function to Gut Bacteria Metabolism of NJT and NJT-75

The choline metabolism is one of the metabolic pathways that is affected by gut microbiota that leads to the formation of trimethylamine (TMA) through host microbial interactions [32]. TMA is subsequently oxidized by flavin monooxygenase enzymes to form trimethylamine-N-oxide (TMAO) in liver, and then TMAO is released into circulation throughout the body [33]. In the present study, the levels of TMAO in urine and serum significantly decreased in the* Realgar* treated group, which indicated the alteration of intestinal flora. Betaine, which is a metabolite of choline, serves as organic osmolytes in biological organisms. Significant raised levels of betaine in urine and serum reflect the enhanced bioavailability of choline to form betaine as a consequence of inhibited degradation pathway from choline to TMAO [11]. In cases where the intestinal microflora has been reduced, the urinary excretion of betaine has been observed to be elevated at the expense of TMAO [34].

Hippurate is formed in hepatic tissue by the conjugation of glycine and benzoic acid, and the formation depends on the favorable supply of ATP [35]. The change in energy metabolism discussed above could lead to reduced levels of ATP and further lead to decreased hippurate [29]. Therefore, hippurate formation is a commonly used clinical marker of liver function and also provides indication of the energy level status [36]. Meanwhile, benzoic acid is primarily synthesized from the intestinal microflora metabolism of aromatic acids or plant phenolics [17]. The reduction of hippurate in urine and serum indicated that the normal balance of gut microflora corresponding to aromatic acid metabolism may be disrupted [37]. Decreased hippurate in urine and serum of* Realgar*-exposed rats also indicated that the balance of intestinal microbial environment was influenced by* Realgar*.

In this study, when* Realgar* was cotreated with NJT and NJT-75, the levels of TMAO, betaine, and hippurate in urine and serum got to the normal standard, which suggested a potential recovery of intestinal environment regulated by NJT and NJT-75.

### 4.4. Regulating Action of Amino Acid Metabolism Disturbance of NJT and NJT-75

Liver is the main metabolic regulatory organ and deeply involved in amino acid absorption, degradation, and metabolism [38], and any hepatic injury might induce amino acids metabolic disturbances [39]. The concentrations of alanine and branched chain amino acids (isoleucine, leucine, and valine) were observed to be decreased in serum from* Realgar* treated rats.

Alanine plays a key role in glucose-alanine cycle between tissues and liver, in which alanine is formed by transamination of glucose-derived pyruvate and is transported to the liver, where its carbon skeleton is reconverted to glucose [40]. The decreased level of alanine in serum from* Realgar* treated animals implied a* Realgar* induced slowdown of glucose-alanine cycle [1]. It has been demonstrated that branched chain amino acids are physiologically important as regulators of the protein and glucose metabolisms [41, 42]. The decreased levels of isoleucine, leucine, and valine that were found in this study suggested that some disorder of the protein and energy metabolisms may be present in animals of* Realgar *group. The increase of urinary amino acids might mean disruption to hepatic amino acid metabolism as well as decline to reabsorb low molecular weight compounds as a typical manifestation of nonspecific proximal tubular damage [43].

Taurine, which is one of the end-products of cysteine catabolism and synthesized mainly in the liver [39], is one of the most abundant intracellular free amino acids and possesses important biological roles [44]. It has a protective action against drug induced toxicity through antioxidant effects [45] and is identified as a liver specific marker of toxicity [46]. In pathologic conditions, the level of taurine would markedly elevate [46]. In this study, the evidently decreased taurine levels after NJT and NJT-75 treatment indicated that they may protect and regulate the metabolic environment to resist the toxic effects of* Realgar*.

Creatine is a nitrogenous organic acid, which is synthesized primarily in liver from the methylation of glycocyamine by S-adenosyl methionine [47]. It is then transported through the blood to the other organs, where phosphorylation occurred [48]. Creatinine is formed from creatine, which is a waste product for the body. Both creatine and creatinine are associated with ATP delivering and consuming processes [47]. It has been reported that both renal and liver injury could lead to the alteration of creatinine [26].

However, when rats were treated with* Realgar* combined with NJT and NJT-75, respectively, these situations mentioned above could be effectively improved.

As_2_S_2_ is insoluble in aqueous solutions, whereas only elements dissolved in gastrointestinal tracts could be absorbed by the body [2]. Inorganic arsenicals, including arsenite (As^III^) and arsenate (As^V^), were reported to be very toxic, with As^III^ being even more toxic than As^V^. Monomethylarsonic acid (MMA) and dimethylarsinic acid (DMA) exhibit only about 1/400 toxicity of inorganic forms, while arsenobetaine (AsB) and arsenocholine (AsC) are almost nontoxic [49].

Since* Realgar* is commonly prescribed in Chinese compound formulae, the amount of arsenic leached from the* Realgar*-containing TCM prescriptions might be influenced by the coexisting components and therefore the arsenic absorption and metabolism might be altered [4]. In vitro studies showed that* Rhei Radix et Rhizoma*,* Scutellariae Radix*,* Platycodonis Radix,* and* Glycyrrhizae Radix et Rhizoma* could reduce the solubility of arsenic to varying degrees in artificial gastric juice or artificial intestine juice [50, 51]. A comparative pharmacokinetic study of the arsenic species in beagle dogs after a single oral administration of* Realgar* alone or NJT showed increased transformation from As^V^ to DMA and faster elimination rate of DMA in NJT, which might be attributed to the drug-drug interactions among the chemical constituents in the compound preparation of NJT [4]. The main components of 75% EtOH fraction of NJT are anthraquinones [52, 53], flavonoids [54–56], and saponins [57–59], which were known from the literatures and by characteristic chromogenic reactions and thin layer chromatographies. Anthraquinones, flavonoids, and saponins could reduce arsenic leached from NJT to a certain extent [50]. All of the findings might be useful for the understanding of detoxification mechanism for* Realgar* in NJT.

## 5. Conclusions

In the present study, a combination of NMR spectroscopic metabonomic analysis, histopathology, and clinical biochemistry assays were used to investigate the active fraction of NJT with toxicity alleviation effect on* Realgar*. Our results indicate that 75% EtOH fraction of NJT has the toxicity alleviation effect on* Realgar*. The obtained results might be useful for scientific understanding of the compatibility mechanisms of NJT. In addition, our work also illustrates that metabonomic approach is a useful tool in the investigation of toxicological effects of traditional Chinese medicines and other xenobiotics.

## Figures and Tables

**Table 1:** Clinical chemistry parameters.

Biochemical parameters	Control group	Realgar group	NJT group	NJT-PET group	NJT-25 group	NJT-50 group	NJT-75 group	NJT-95 group
AST (U/L)	106.24 ± 15.68	153.50 ± 29.27^*∗*^	118.00 ± 16.53	127.80 ± 20.92	138.80 ± 32.55	125.37 ± 12.26	120.39 ± 16.28	132.34 ± 25.58
ALT (U/L)	17.66 ± 3.01	39.67 ± 7.89^*∗*^	20.54 ± 5.21	26.11 ± 7.14	32.47 ± 4.56^*∗*^	19.21 ± 2.12	22.69 ± 6.08	33.76 ± 8.13^*∗*^
ALP (U/L)	111.29 ± 21.05	168.01 ± 37.14^*∗*^	123.17 ± 29.70	145.56 ± 18.93^*∗*^	118.85 ± 17.22	137.41 ± 15.12^*∗*^	105.96 ± 13.80	121.61 ± 20.45
BUN (mmol/L)	7.40 ± 0.68	12.33 ± 0.80^*∗*^	8.12 ± 1.25	8.44 ± 0.86	7.79 ± 0.53	8.64 ± 1.41	8.00 ± 0.75	10.81 ± 1.88^*∗*^
CREA (*μ*mol/L)	15.76 ± 1.96	31.13 ± 4.02^*∗*^	16.11 ± 2.05	25.07 ± 5.62^*∗*^	19.00 ± 3.82	18.54 ± 3.49	16.08 ± 2.55	15.01 ± 0.97
TG (mmol/L)	0.49 ± 0.06	0.53 ± 0.05	0.55 ± 0.11	0.50 ± 0.04	0.60 ± 0.28	0.46 ± 0.17	0.57 ± 0.10	0.49 ± 0.05
TC (mmol/L)	1.22 ± 0.16	1.11 ± 0.28	1.51 ± 0.36	1.23 ± 0.23	1.44 ± 0.54	1.30 ± 0.19	1.30 ± 0.31	1.16 ± 0.27

*Statistics.* ^*∗*^ *P* < 0.05 versus control group. Data are presented as mean ± SD of six animals per group.
